# Rheumatology and Its Inception in the South West

**Published:** 1992-08

**Authors:** George D. Kersley


### Rheumatology and its inception in the South West

**George D. Kersley, O.B.E., T.D., D.L., M.D., D.Sc., F.R.C.P.**

The first chapter in the history of rheumatology, apart from the foresight of a few great physicians like Guillaume de Baillou 1538-1616, was produced by Thos Sydenham, who wrote an amazing description of acute rheumatism in 1676. This was followed up by Pitcairn linking it with cardiology in 1788 and Archibald Garrod 1817-1907 on gout. Much of our early knowledge comes from books and treatises of the Spa doctors researching the "waters". Examples are George Cheyne in 1720 on gout, Caleb Hillier Parry, whose most important thesis was in 1786 on thyrotoxicosis, Kent Spender in 1887 on osteoarthritis and Gilbert Bannatyne in 1896 on rheumatoid arthritis, who also gave the first description of its xray diagnosis in 1911.

Serious interest in rheumatology started in the 1920's, with Alison Glover's Reports on its incidence(1924-1928). This led to the setting up of the Royal College of Physicians' Committee in 1932 and this was followed in 1936 by the formation of the Empire Rheumatism Council, now the ARC. In the same year the Heberden Society was formed.

The first consultant physician to make the subject his specialty was Will Copeman, who qualified at St Thomas' Hospital in 1925 and who moved to the Middlesex to specialise in rheumatology. In 1932, having just taken my Membership, I realised that I would not have been dismayed if confronted with a question on some rare brain tumour but I would have felt a question on rheumatoid arthritis was unfair! As a very junior physician, there was no chance of working in the field of rheumatology at Barts. To the disgust of Professor Sir Francis Fraser, I decided to come to Bath with support only from Lord Horder, always twenty years ahead of his time. I must admit however that Fraser changed his mind and wrote the Preface to my first small book on the subject.

At that time, throughout the world, the Spas were the only centres where "rheumatic" cases were treated and our only hospitals were at Bath, Buxton and Harrogate. At Bath there was also probably the best non-teaching general hospital in the country - the Royal United Hospital (RUH).

The Royal Mineral Water Hospital, dating from 1742, was pretty archaic and still taking cases of gall stones in order that the "waters" might wash away the stones! Most of the physicians were to me very elderly and mainly interested in private work and the general medicine at the RUH. They allowed a young firebrand, with the support of Vincent Coates, to recruit Barnes Burt and Leslie Hill from Buxton. By 1936 by Act of Parliament, we changed the hospital's name to the Royal National Hospital for Rheumatic Diseases, and it became the first hospital in the world to devote itself solely to rheumatology and to use any treatment known to be helpful in its treatment. Charles Kindersley introduced, for instance, manipulation and serial plaster splintage to reduce deformities and carried out, at my behest, the first two disc operations performed in this country. There were great plans for a new much larger hospital on the site now occupied by the Technical College, but this was aborted by the onset of the War. Being already in the Territorial Army, I missed the "excitement" of the bombing of the South West corner of the hospital and came back to the fight to prevent the dissolution of the hospital. As the Chairman of the Medical Board, I was faced with a five years fight with the Ministry not to accept a few wards at the RUH as its replacement.

During the War, Rheumatology took over Physical Medicine and Rehabilitation. After the War was over, a "war" broke out between the two specialties. Rheumatology, helped by the research arising from the discovery of cortisone by the rheumatologist, Phil Hench, was at last accepted as a respectable specialty. It was however afraid of being dragged down by association with the less well qualified and newer Physical Medicine specialty. This breach has now been healed and most hospitals devoted to rheumatology, now have important sections devoted to rehabilitation.

In 1948, when the National Health Service came into being and we were for the first time paid for our hospital work, I was asked whether I wished to give up General Medicine and take on other duties in Rheumatology, in particular to start a regional research centre at the RNHRD and work also at the Bristol Royal Infirmary and Southmead Hospital. This was a difficult decision but I accepted. I was also made a Senior Lecturer in Physical Medicine at Bristol University and given a small advisory post at Oxford.

Bath and Bristol had always been rivals and Professor Bruce Perry was not keen on specialisation, except in his own interest, cardiology. The psychiatrist and rheumatologist were an anathema to him. In Bristol he was all powerful and I often found myself a minority of one in committee arguments. I was the first medical Bathonian to have an appointment at the Teaching Hospital. I enjoyed my work in Bristol but I was happier when Stephen Merivale asked me to move my clinic from the BRI to the Bristol General.

The rift between Bath and Bristol in non-medical spheres was such that there was surprise when I invited the Lord Mayor to take the salute at the Bath Tattoo.

The first undergraduate link with Bath was when Professor Wilkinson of Birmingham asked me to organise a day's practical demonstrations each year at the RNH RD and shortly afterwards Professor Perry allowed me to arrange a similar day for the final year Bristol students - each day finished up with a little drinks party at home.

After this a closer link was established when Malcolm Jayson, now Professor of Rheumatology at Manchester, was given a joint appointment with Professor Perry's staff at the RNHRD. This was followed by a similar arrangement for Paul Dieppe, who shortly afterwards in 1987, to our delight, was promoted to the ARC Chair at Bristol. There he has formed a strong team, including John Kirwan as Senior Lecturer, with a special interest in osteoarthritis and crystal osteopathies.

Now in 1988 Peter Maddison has become Glaxo Professor for the study of osteoarticular pathology, to include osteoporosis.

It has taken some fifty years for Rheumatology to mature from being a suspect outcast to becoming one of the most rapidly expanding specialities, leaning heavily on the sciences, such as genetics and immunology, to cover perhaps the greatest and most expensive group of disabling diseases.

